# Applications of Traditional Herbal Ingredients in Skincare: Mapping the Research Landscape and Innovation Trajectories Over Four Decades

**DOI:** 10.1111/jocd.70363

**Published:** 2025-07-21

**Authors:** Kexin Deng, Yi Liu, Dian Li, Can Liu

**Affiliations:** ^1^ Department of Plastic and Reconstruction The Third Xiangya Hospital of Central South University Changsha Hunan Province China; ^2^ Department of Epidemiology UCLA Fielding School of Public Health Los Angeles California USA

**Keywords:** healthy skin, herbs, plants, skin care

## Abstract

**Objective:**

Along with economic and technological advancements, traditional herbal ingredients have garnered increasing attention in skincare due to their antioxidative properties, low toxicity, multitargeted effects, and broad cultural acceptance. However, there remains a lack of comprehensive knowledge frameworks and systematic research trend analyses in this field. This study examines the scientific landscape and innovation patterns of herbal skincare, aiming to establish a framework for integrating traditional wisdom with modern dermatological science.

**Methods:**

A systematic literature search was conducted in the Web of Science Core Collection (SCI‐E). CiteSpace, VOSviewer, and ArcGIS were employed to analyze leading contributors, research trends, and thematic evolutions.

**Results:**

The analysis yielded 1593 papers authored by 753 researchers from 507 institutions across 102 countries. China emerged as the top contributor, while Industrial Crops and Products was the most highly cited journal, and Zengin, Gokhan, was the most prolific author. Geographical clustering and cultural convergence characterized the distribution of countries, institutions, and authors. Keyword clustering identified 20 research networks, with recent clusters including #6 “molecular docking,” #7 “chromatography‐mass spectrometry,” and #15 “mixture design.” Persistently trending keywords included “Mechanism,” “Gut microbiota,” “Network pharmacology,” and “Association.”

**Conclusion:**

Research on herbal skincare has evolved from raw plant usage to advanced refinement techniques, spanning animal, cellular, and molecular studies, including high‐throughput screening approaches. Formulations have progressed from complex mixtures to single‐compound extractions and bioactive monomers. A comprehensive research system has been established, yet standardization in dosage, application, and manufacturing remains crucial. Future studies should prioritize multicenter clinical trials and evidence‐based validation. This study highlights the transition from empirical herbal applications to precision skincare, advocating for global collaboration between traditional medicine and modern regulatory science.

## 1 Introduction

The skin is the largest organ and primary defensive barrier of the human body, performing vital physiological functions including thermoregulation, maintenance of metabolic homeostasis, and protection against environmental insults [1]. Given its visible nature, skin conditions significantly influence social perception and interpersonal dynamics [2]. Empirical evidence indicates that individuals with youthful skin characteristics often exhibit enhanced social confidence and reduced psychological stress during interpersonal interactions [3]. Driven by these psychological demands and the non‐invasive nature of topical skincare, daily skincare has become an indispensable sociocultural practice and a health necessity for urban populations worldwide [4].

The historical trajectory of skincare innovation parallels advancements in human technology. Archaeological evidence from ancient civilizations reveals the early use of plant extracts to protect and enhance the skin [5]. With the Industrial Revolution (17th–19th centuries) and contemporary biotechnological progress, the definitions and compositional systems of skincare products have grown increasingly sophisticated [6]. This evolution has transformed skincare from gender‐specific luxury items into daily essentials for all demographic groups, including male consumers and pediatric populations [6, 7, 8]. However, mainstream modern skincare products face limitations such as ingredient irritancy, environmental toxicity, singular mechanisms of action, and hormonal dependency. Herbal skincare products address these issues through natural polyphenols, terpenoids, and other compounds that enable multitarget regulation while improving biodegradability, thereby balancing efficacy and ecological safety [9, 10, 11].

Herbal ingredients have become critical resources in this field, with a growing number of bioactive phytochemicals being incorporated into cosmetic formulations. Ancient medical texts such as the *Huangdi Neijing* (The Yellow Emperor's Inner Classic, 475–221 bc) and *Shennong Bencao Jing* (Divine Farmer's Materia Medica, Han Dynasty) document over 200 plant‐based medicines with dermatological applications [12]. The *Zhouhou Beiji Fang* (Jin Dynasty) systematically categorized 33 herbal formulas for cosmetic purposes, such as the Qi Bai San formulation for skin brightening. Modern scientific validation has converted these historical insights into commercialized active ingredients, including camellia oil, aloe polysaccharides, and ginsenosides [5, 13, 14, 15].

Current skincare strategies focus on five core objectives: photoprotection, anti‐aging, hydration, pigmentation correction, and anti‐irritation [16]. Most plant‐derived active components naturally possess properties to repair and protect the skin from environmental pollution, chemical exposures, atmospheric temperature fluctuations, and UV radiation, making them preferred sources of photoprotective agents [17, 18]. The use of sunscreens to prevent premature skin aging and skin cancer represents a simple and cost‐effective approach [11]. Notably, studies suggest that cosmetics containing Chinese herbal ingredients are more suitable for highly sensitive skin, as traditional herbs or their active compounds reduce intracellular ROS levels, demonstrating potent antioxidant properties with particular advantages in anti‐aging and anti‐photoaging [19, 20]. The efficacy of herbal products and specific phytochemicals in mitigating aging has been scientifically validated [21]. Representative examples of well‐researched botanicals include aloe, ginseng, and saffron [19, 22]. Natural herbs meet the multifunctional demands of skincare due to their proven efficacy, multitarget mechanisms, and favorable safety profiles.

While medicinal herbs demonstrate advantages in skin brightening and antioxidant effects, their oral administration presents narrow safety margins, particularly concerning hepatorenal toxicity, gastrointestinal complications, and hypersensitivity reactions [23, 24]. For example, *Angelica dahurica* effectively inhibits melanin synthesis and promotes keratinocyte proliferation but can induce photosensitive liver injury and renal tubular necrosis when ingested orally [25, 26]. Topical application through surface or epidermal‐layer delivery offers a safer alternative by reducing systemic exposure and avoiding toxicity. Market analyses indicate exponential growth in herbal cosmeceuticals, with over 400 specialized manufacturers operational by 2011 and a projected compound annual growth rate (CAGR) of 14.3% through 2030 [27]. Nevertheless, significant challenges remain regarding standardized protocols for product development and clinical application, regulatory oversight, and evidence‐based guidelines.

Bibliometric analysis provides a robust framework for quantitative mapping of scientific fields through multilevel assessments of contributing entities (countries, institutions, and authors) and the evolution of knowledge structures [28]. This study employs research structure visualization and temporal evolution analysis via CiteSpace and VOSviewer to:

1. Trace the historical development of herbal skincare research (1981–2024)
2. Identify key contributors and collaborative networks across national, institutional, and author levels, along with major journals in the field.
3. Describe the research hotspots and trends in the field, summarize the compositional framework of the field's themes, as well as the macroscopic historical progress.
4. Propose novel research directions and recommendations for advancing the discipline and solving challenges.

## 2 Method

### 2.1 Research Methods

As a methodological framework rooted in quantitative science, bibliometrics uses metadata attributes of scholarly publications (e.g., citations, authorship patterns) as analytical units [29]. This discipline applies statistical methodologies to systematically characterize, evaluate, and predict the trajectory of development within the scientific field, thus serving as a predictive tool for technological evolution [29]. Its analytical strength lies in deciphering the implicit conceptual architectures embedded in academic discourse, particularly through computational examination of semantic elements such as terminological clusters and citation networks [30]. In addition, these techniques help synthesize fragmented research findings into coherent maps of knowledge, enhancing interdisciplinary understanding of specialized fields through spatial–temporal data visualization [31].

This study was visualized using CiteSpace and VOSviewer [32, 33], and a detailed description of the software principles and definitions of terms can be found in a Supporting Information.

### 2.2 Data Resource

This paper selects the Web of Science Core Collection (WoSCC). The index is the Science Citation Index Expanded (SCIE). The retrieval formula of this paper is as follows:(TS = (“herb*” OR “tradit* medic*” OR “chine* Medic*” OR “plant*” OR “Phytoth*” OR “natur*” OR “Organic*”)) AND TS = (“skincare*” OR “skin* care*” OR “Skin* health*” OR “skin* treatment*” OR “skin‐care*” OR “cosmet*”). All electronic searches were performed on January 1, 2025, in China. A total of 16 730 articles were searched, and 12 509 original articles were selected and written in English. We subsequently conducted a triple‐blind screening of the initially included literature based on the following inclusion and exclusion criteria. To ensure the accuracy and relevance of the literature, we used the intersection of the literature selected by the three reviewers for the subsequent analysis. The three reviewers are orthopedic PhDs from three different provinces in China.

**FIGURE 1:**
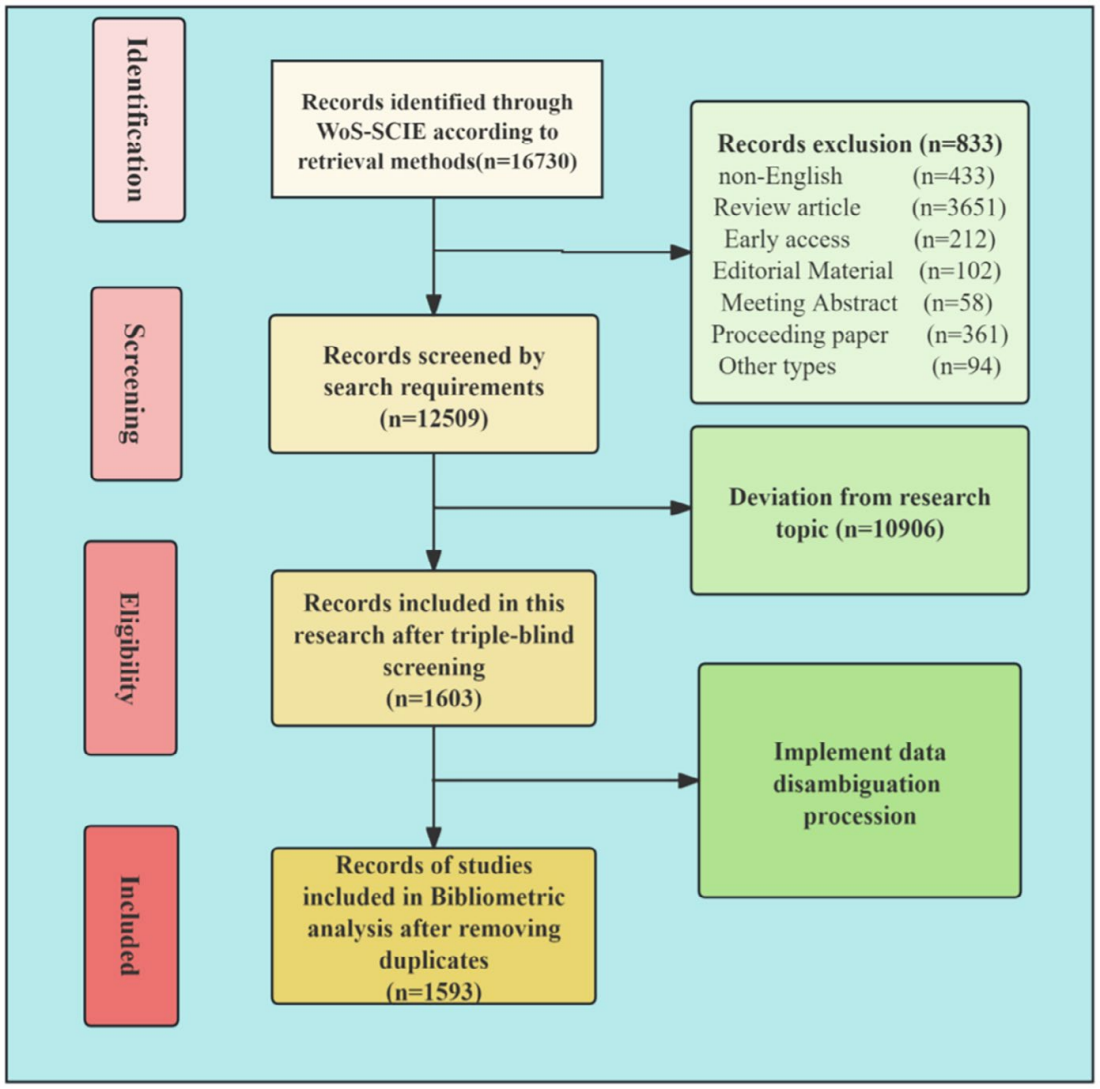
A search strategy was used for bibliometric analysis by PRISMA.

Finally, after eliminating duplicate articles, this paper carries out a bibliometric analysis of 1593 literature. The whole process of using PRISMA for literature search and screening is shown in Figure 1.

### 2.3 Inclusion Criteria

1. The search is limited to publications that are written in English and have complete reference and citation records.
2. The main content and purpose of the study focus on skincare and promoting skin health, including clinical human trials, animal experiments, cellular experiments, molecular biology experiments, bioinformatics analysis, component analysis, testing, etc.
3. The research content is closely related to natural plant‐derived ingredients, with study materials including raw plant materials, metabolites, coarsely processed materials, finely processed products, extracts in various solvent forms, active substances, poly‐herbal or mono‐herbal formulas, etc.
4. The research also covers various application methods of natural herbal ingredients, such as topical use, oral administration, intravenous injection, microneedling, etc.

### 2.4 Exclusion Criteria

1. Non‐English literature and articles with incomplete references or citations.
2. Articles that incidentally mention keywords related to natural herbs but involve study objectives or research materials unrelated to natural herbs or their derivatives will be excluded. For example: “There are numerous treatments for acne, including herbal products, antibiotics, and chemical peels. Our study aims to investigate the efficacy of probiotic products in acne treatment.”
3. Articles that incidentally mention keywords related to skincare but focus on research topics unrelated to skin health or related fundamental studies will be excluded. Example: “Curcumin not only improves skin health but also treats inflammatory bowel disease (IBD). Our study aims to explore the role of curcumin in Crohn's disease….”
4. Articles lacking substantive research, where conclusions are merely speculative or discussed in the results/discussion sections without supporting evidence, will be excluded. Example: “Given its antioxidant properties, we hypothesize/speculate that it may play a significant role in skincare.”
5. Studies solely related to cosmetic purposes without relevance to skin health will be excluded.

## 3 Result

### 3.1 Analysis of Publishing Trend

**FIGURE 2:**
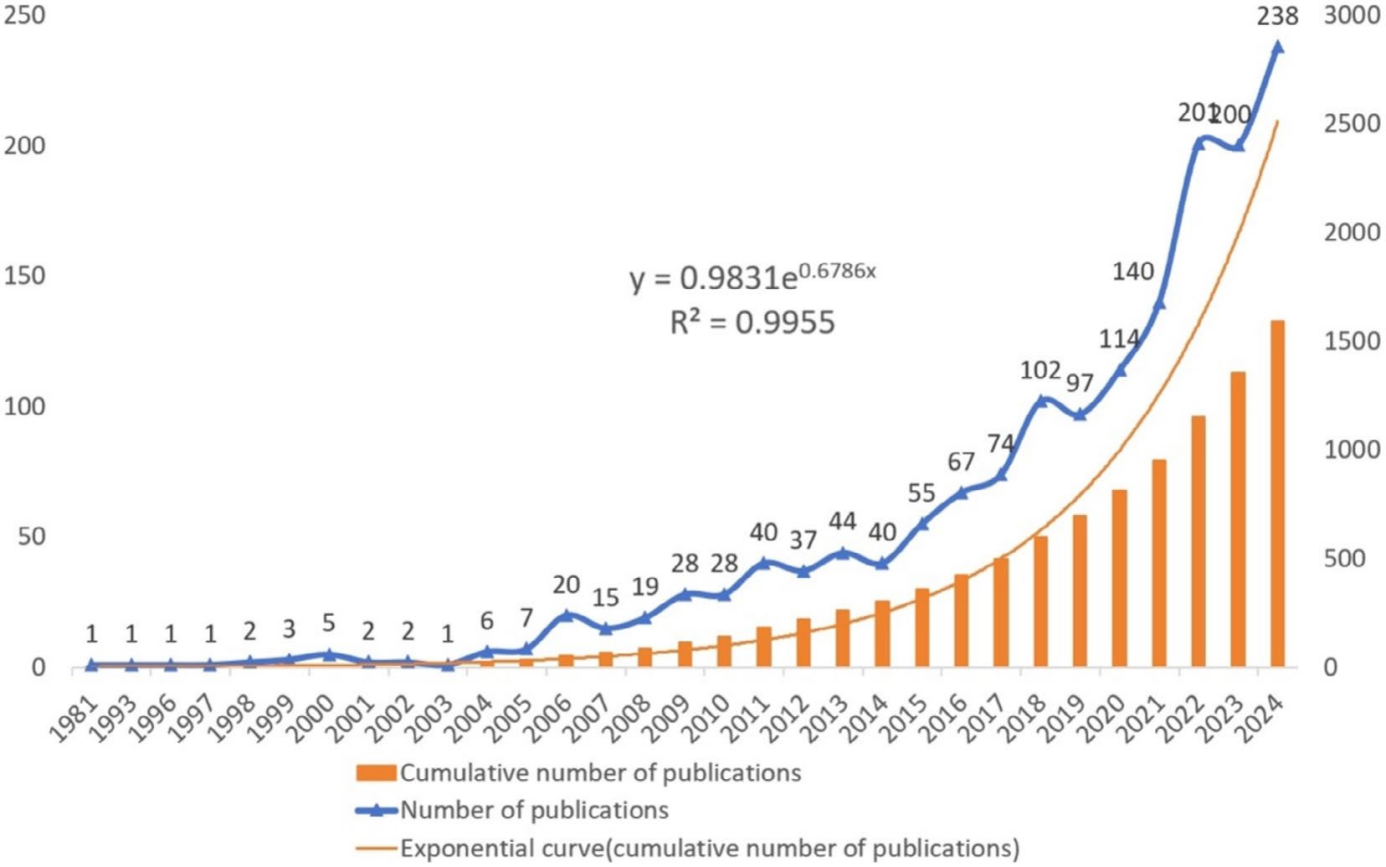
Annual publication trends in herbal skincare research (1981–2024).

The publication trend of literature is an important index reflecting the research development in a certain field. Therefore, by drawing the literature quantity‐time curve, we can effectively evaluate the research status in this field and predict the future development trend. Figure 2 shows the annual distribution of publications related to the use of traditional herbs for skin care. The bibliometric analysis included 1593 publications.

From 1981 to 2005, the annual number of publications remained relatively low, with fewer than 10 articles published each year. This reflects the early developmental phase of research on traditional herbal therapies for skincare. From 2006 onward, however, publication output began to steadily increase, signaling growing interest in the significance of herbal‐based skincare studies. Between 2017 and 2019, there was a marked upward trend in publications, illustrating both the expansion of the research community and a heightened focus on validating traditional herbal applications. By 2024, the field reached its peak, with nearly 238 papers published in a single year, marking the culmination of this trajectory and demonstrating heightened recognition and broader acceptance of traditional herbs in modern skincare and beauty practices.

Overall, this trend highlights not only quantitative growth in research output but also an expanding recognition of the field's importance. The sharp rise in publications in recent years underscores that herbal skincare is gaining credibility and momentum within academia. Promoting international collaboration and cross‐disciplinary research will be critical to advancing this domain further and ensuring its sustained growth and impact.

### 3.2 National, Institutional, Author, and Journal Analysis

#### 3.2.1 National Analysis

**FIGURE 3:**
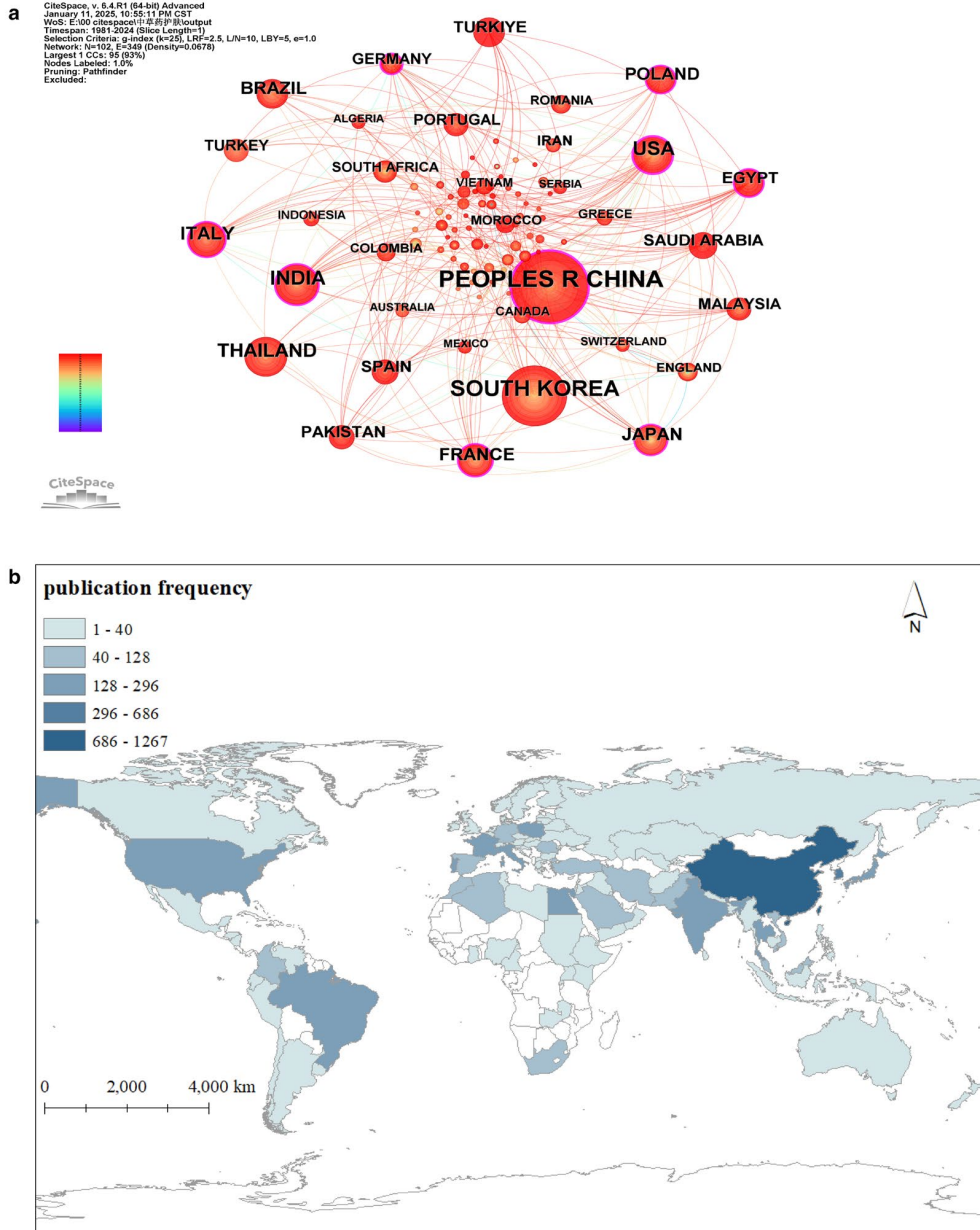
Country‐level publication analysis. (a) International collaboration network. The network spans 1981–2024 (1‐year intervals). Nodes denote countries, scaled by publication counts. Purple outlines highlight nations with intermediary centrality > 0.1, reflecting their brokerage role in cross‐border collaborations. (b) Geospatial publication density, geographic distribution mapped via ArcGIS, with node color intensity proportional to national publication output (darker hues = higher productivity).

Figure 3a and Table 1 present a national analysis with 102 network nodes (count of the total country) and 349 connections (count of collaborations), with a density of 0.0678. The People's Republic of China (Peoples R China) leads globally in the number of publications on traditional herbal skincare treatments, far exceeding other nations. This dominance stems from China's historical roots and thriving development of herbal medicine, supported by substantial government funding dedicated to traditional herbal research. These efforts have established a large‐scale, systematic research framework. The centrality metrics reveal that China not only holds a pivotal position in traditional herbal skincare studies but also actively drives international academic collaboration. The widespread cultural promotion of herbal concepts and their integration into daily life in China has fostered high public acceptance of herbal‐based skincare products. These factors collectively position China as the international frontrunner in this field, contributing the most significant scholarly output and practical advancements.

**TABLE 1:** The frequency and centrality of publication in countries/regions.

Country	The first publication year	Frequency	Centrality
People's Republic of China	2005	321	0.24
South Korea	2001	221	0.07
India	1998	118	0.15
USA	1996	112	0.27
Thailand	2007	98	0.02
Italy	2000	83	0.17
Brazil	2008	70	0.05
France	2002	68	0.17
Japan	1993	68	0.1
Poland	2010	58	0.1
Pakistan	2005	48	0.08
Spain	2006	44	0.1
Türkiye	2023	44	0.07
Saudi Arabia	2016	44	0.04
Egypt	2015	44	0.15
Malaysia	2010	39	0.08
Portugal	2013	36	0.03
Turkey	2006	36	0.02
Germany	2000	36	0.15
Iran	2008	29	0

Comparatively, South Korea ranks second in the contribution of the number of studies in this field. However, the centrality approach to zero indicates that its research tends to be conducted independently, which means limited academic communication with other countries. The lack of international exchange may weaken the credibility and innovativeness of the research. In addition, including China, countries and regions such as *India* (*INDIA*), the *United States* (*USA*), Italy, France, Japan, and Poland have centrality values greater than 0.1, suggesting that these countries have significant international collaborations and play important roles in the academic research network. Additionally, as shown in Figure 3b, among the top 10 countries in terms of research output, most are concentrated in Asia, particularly East Asia, highlighting the regional identity of research on traditional herbal skincare.

#### 3.2.2 Institutional Analysis

**FIGURE 4:**
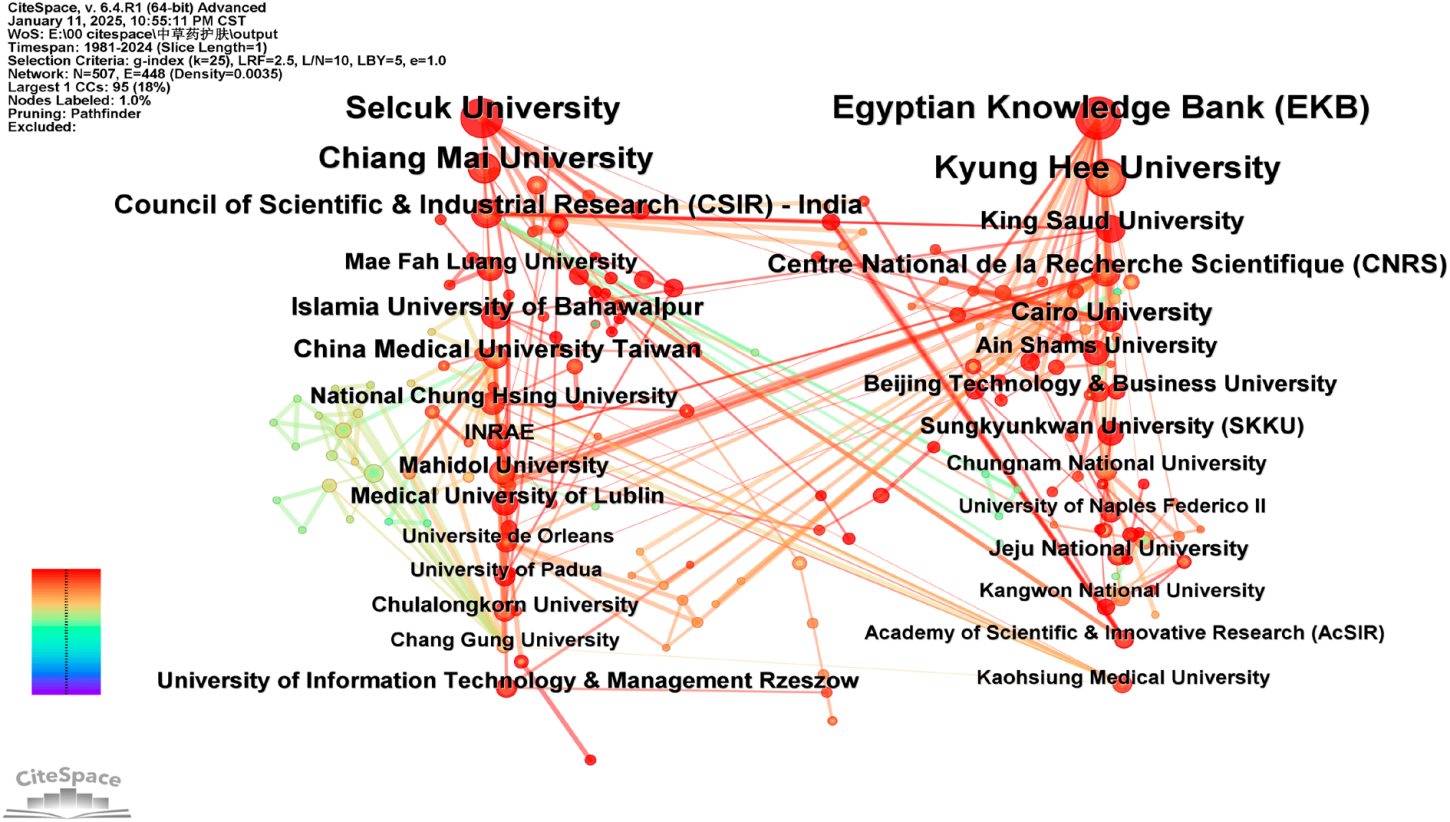
Institutional co‐occurrence map. Nodes represent academic institutions, with size proportional to publication output. Purple‐ringed nodes denote entities exhibiting high brokerage potential (betweenness centrality > 0.1) within the cooperative landscape.

As shown in Figure 4 and Table 2, using “Institutions” as the analytical focus in CiteSpace, we obtained an analysis graph of institutions with 507 network nodes, 448 connections, and a density of 0.0035.

**TABLE 2:** The top 15 institutions of publication.

Institution	The first publication year	Frequency	Centrality
Egyptian Knowledge Bank (EKB)	2015	41	0.05
Kyung Hee University	2011	38	0.04
Selcuk University	2018	34	0.04
Chiang Mai University	2007	32	0.04
Chinese Academy of Sciences	2005	25	0
Council of Scientific & Industrial Research (CSIR), India	2006	19	0.05
Centre National de la Recherche Scientifique (CNRS)	2013	18	0.05
Islamia University of Bahawalpur	2010	16	0.01
China Medical University, Taiwan	2006	15	0.04
Cairo University	2016	15	0.05
King Saud University	2023	15	0.1
Konkuk University	2017	13	0
Beijing Technology & Business University	2015	12	0
Ain Shams University	2021	12	0.01
Medical University of Lublin	2022	12	0.03

The Egyptian Knowledge Bank (EKB) ranks as the institution with the highest output of publications on skincare and traditional herbal medicine, having published 41 papers. Notably, eight of the top 10 institutions by publication count exhibit centrality above 0.04, indicating a relatively higher degree of inter‐institutional collaboration within the field. This collaborative trend likely stems from the multidisciplinary nature of herbal skincare research, which spans pharmacology, phytochemistry, dermatology, bioengineering, network pharmacology, and mass spectrometry, necessitating extensive cross‐disciplinary cooperation.

However, institutions such as the Chinese Academy of Sciences and Centre National de la Recherche Scientifique (CNRS)—despite their high publication output—have centrality scores of 0, reflecting a preference for independent research with limited collaboration. The absence of institutions with betweenness centrality exceeding 0.1 suggests that dominant hubs facilitating collaborative networks are lacking, potentially hindering the field's expansion. Established high‐output institutions typically serve as collaborative nuclei, enabling newer organizations to enhance research capacity through network integration.

A striking regional pattern emerges: nearly all top 20 institutions are based in East Asia, particularly China, underscoring the field's geographical concentration.

#### 3.2.3 Authors Analysis

**FIGURE 5:**
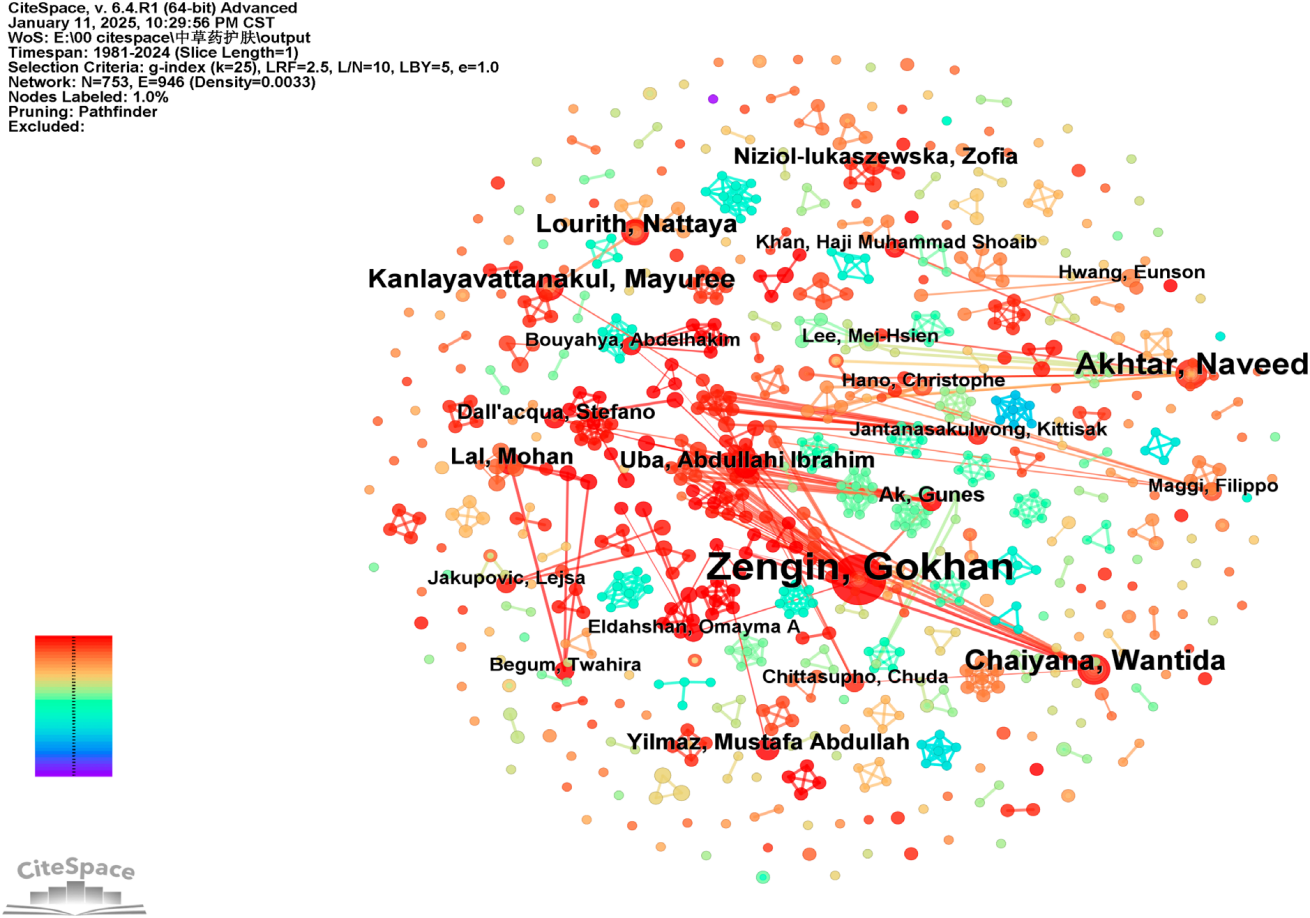
Author‐centric network mapping in CiteSpace constructed co‐authorship relationships, where node size is scaled by individual publication output.

According to CiteSpace author analysis, the network comprises 753 nodes and 946 connections in Figure 5. As shown in Table 3, Zengin, Gokhan, emerged as the most prolific author in the field of herbal skincare, with 33 publications. However, despite the large number of researchers involved, collaboration among researchers remains sparse, as indicated by centrality values close to zero, reflecting a high degree of research independence. Only three authors have published more than 10 articles in this domain, underscoring the lack of extensive interpersonal collaboration in studying the intersection of skincare and traditional herbs.

**TABLE 3:** Top 10 authors in publication count.

Authors	The first publication years	Frequency	Centrality
Zengin, Gokhan	2018	33	0
Akhtar, Naveed	2010	14	0
Chaiyana, Wantida	2020	11	0
Kanlayavattanakul, Mayuree	2017	9	0
Lourith, Nattaya	2017	8	0
Lal, Mohan	2023	6	0
Yilmaz, Mustafa Abdullah	2024	6	0
Uba, Abdullahi Ibrahim	2024	6	0
Niziol‐Lukaszewska, Zofia	2018	6	0
Dall'acqua, Stefano	2021	5	0

Limited collaboration may stem from traditional knowledge transmission practices in herbal medicine, which often rely on mentor‐apprentice relationships rather than systematic collaborative frameworks. Furthermore, some researchers prioritize the independence or confidentiality of herbal formulations within their academic lineages, making cross‐team collaboration more challenging. This lack of cooperation critically hinders progress and innovation in the field. Enhancing collaboration among authors and fostering international partnerships could be pivotal strategies to advance future research and overcome existing barriers.

#### 3.2.4 Journal Co‐Citation Analysis

**FIGURE 6:**
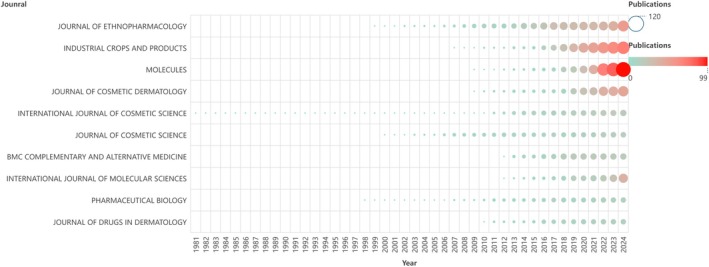
Cumulative graph of the number of published papers in the first 10 years of the journal. The vertical axis represents the cumulative number of papers published in the journal, and the horizontal axis represents the publication time. The redder the color and the larger the node, the greater the cumulative number of papers published.

In Figure 6 and Table 4, *Industrial Crops and Products stands* as the most‐cited journal in this field, with 1907 citations, highlighting its pivotal role in advancing research on herbal applications in skincare. Its prominence reflects both its pioneering contributions and the scholarly emphasis on plant‐derived innovations.

**TABLE 4:** Journals with the top 10 citation numbers.

Rank	Journals	Citations
1	Industrial Crops and Products	1907
2	Journal of Ethnopharmacology	1720
3	Molecules	1186
4	Journal of Photochemistry and Photobiology B‐Biology	749
5	EMBO Journal	716
6	Journal of Cosmetic Dermatology	604
7	Biochemical Society Transactions	583
8	International Journal of Molecular Sciences	543
9	BMC Complementary and Alternative Medicine	508
10	Phytomedicine	434

*Journal of Ethnopharmacology* (1720 citations) and *Molecules* (1186 citations) rank second and third, respectively. These journals, along with Industrial Crops and Products, have each published over 50 relevant articles, demonstrating their quantitative and qualitative dominance. Notably, *EMBO Journal* and *Biochemical Society Transactions*—despite publishing only one article each—secured positions in the top 10 due to their groundbreaking findings, underscoring the impact of high‐prestige journals in shaping the field.

Journals focusing on chemistry and biomolecular themes, such as the I*nternational Journal of Molecular Sciences* (543 citations) and *Molecules*, have significantly contributed to the field's breadth. Journal of Photochemistry and Photobiology B: Biology also ranks among the most influential, bridging light‐related biochemistry and skincare research and amplifying its reach across broader scientific communities.

Specialized journals like *BMC Complementary and Alternative Medicine, Journal of Cosmetic Dermatology*, and *Biochemical Society Transactions* gained recognition for their unique perspectives on herbal skincare, particularly in areas such as traditional medicine integration, cosmetic science, and translational research. The journals co‐citation network reveals that research hotspots cluster around pharmacology, biochemistry, cell biology, product development, and alternative medicine. This multidisciplinary convergence highlights the complexity and interdisciplinary nature of herbal skincare studies, where advances rely on integrating diverse expertise to address both scientific and practical challenges.

### 3.3 Keywords Cluster, Keywords Burst, and Frontiers Analysis

#### 3.3.1 Keywords Clusters

**FIGURE 7:**
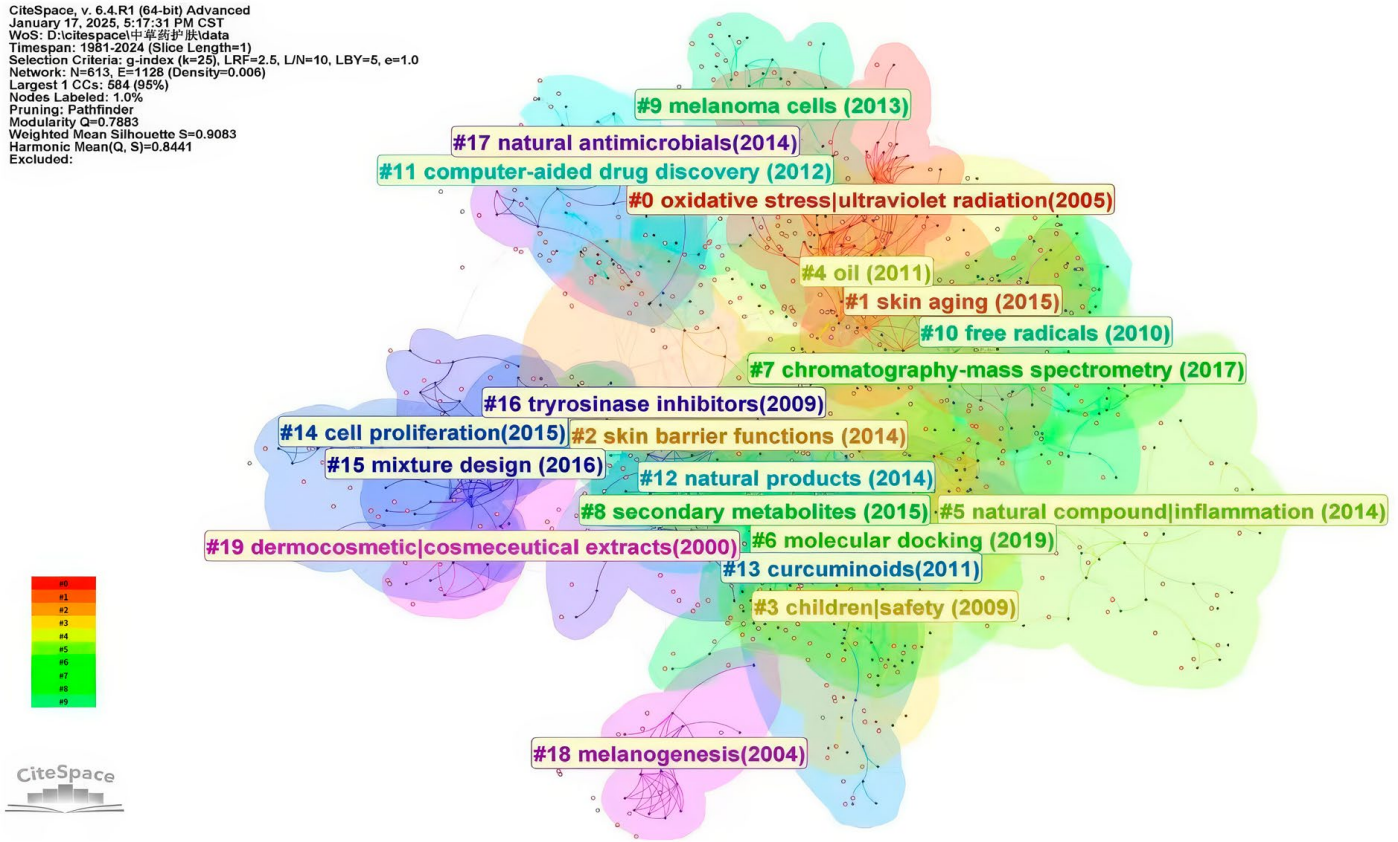
Keywords cluster analysis selected the “cluster” option and used the pathfinder algorithm to draw the connection line, so as to ensure the rationality of the cluster classification. A total of 20 keyword clusters were identified, with each cluster assigned a different color according to the timestamp in the bottom left corner. The names of the clusters were derived from a set of representative keywords with the help of the LLR algorithm.

These clusters are as follows: Figure 7: #0 oxidative stress|ultraviolet radiation (2005), #1 skin aging (2015), #2 skin barrier functions (2014), #3 children|safety (2009), #4 oil (2011), #5 natural compound|inflammation (2014), #6 molecular docking (2019), #7 chromatography‐mass spectrometry (2017), #8 secondary metabolites (2015), #9 melanoma cells (2013), #10 free radicals (2010), # 11 computer‐aided drug discovery (2012), # 12 natural products (2014), # 13 curcuminoids (2011), # 14 cell proliferation (2015), # 15 mixture design (2016), #16 tyrosinase inhibitors (2009), # 17 natural antimicrobials (2014), #18 melanogenesis (2004), and # 19 dermocosmetic|cosmeceutical extracts (2000).

Through identifying clustered keywords and analyzing them chronologically, we observed that Cluster #19 had the earliest average timeline, with keywords emerging around 2000. Early research primarily focused on the discovery of skincare plants and crude extraction methods of active components (e.g., water extracts and alcohol extracts). Initial studies at the animal level explored melanin inhibition and UV protection, including UV absorption and preventing skin damage (linked to Cluster #0). Later research shifted toward cellular models and enzymatic production processes. Cluster #18, with an average keyword emergence year of 2004, centered on natural herbal ingredients' interference with melanin production. Besides, research on melanogenesis persisted throughout the field, with extensive studies on melanin‐producing cellular models and enzymatic pathways also appearing in Clusters #19 and #9 (average years: 2009 and 2013).

A key feature of traditional herbs and their active compounds is their superior antioxidant and free radical scavenging capabilities. In skincare, antioxidant efficacy has been emphasized early on and remained a consistent focus, as shown in Cluster #0 (2005) to Cluster #10 (2010), where studies on oxidative stress evolved from cellular models to established animal‐level systems.

Around 2009, the role of herbs in dermatitis management, a permanent subject in children (e.g., eczema and atopic dermatitis) had been extensively studied and validated in clinical practice and animal studies [34]. Additionally, safety concerns in herbal therapy and skincare have been emphasized and prioritized, as represented by Cluster #3. More nuanced issues, such as skin barrier function research, became prominent in Cluster #2, averaging around 2014. Around 2011, curcumin emerged as a representative active compound from natural plants, attracting extensive research interest. Plant essential oils also gained global scholarly attention for their role in skin health.

Clusters #11, #7, and #6 (average years: 2012, 2017, 2019) focused on advancements in network pharmacology database development, molecular component analysis via mass spectrometry, and related methodologies. These approaches have enabled researchers to rapidly explore the complex interactions between multiple compounds in herbal formulations and biological targets relevant to skincare [35]. From a systemic perspective, herbal formulations provide a comprehensive strategy for synergistically regulating multiple pathways—such as inflammation, oxidative stress, UV protection, and melanin inhibition—through their diverse components [35, 36, 37]. Around 2014, studies surged on the antimicrobial effects of natural herbs against skin pathogens like *Cutibacterium acnes* [38] and *Staphylococcus epidermidis* [39], as well as their role in balancing human skin microbiota [40].

Research on herbal ingredients for combating skin aging is highlighted in Cluster #1, with keywords like hyaluronidase [41], elastase [42], and elastase inhibitor, focusing on mechanisms of natural actives against aging‐related enzymes. Cluster #14 includes skin aging models such as 3T3 and HaCaT cells, alongside studies on herbal compounds for skin cancer prevention [43].

#### 3.3.2 Keyword Outbreak Analysis

In this study, we applied the Bursts detection algorithm in CiteSpace to visualize the evolution of research hotspots related to skincare and traditional herbal medicine within the Web of Science database, shown in Table 5. The generated map presents the top 50 keywords, illustrating their burst intensity and duration. Currently, the most significant keywords include “caenorhabditis elegans,” “rosmarinic acid,” “economic growth,” “nanoparticles,” “assisted extraction, ‘formulation,’ and ‘acetylcholinesterase.’”

**TABLE 5:** Top 50 keywords with the strongest citation bursts.

Keywords	Year	Strength	Begin	End
Ultraviolet radiation	1999	2.17	1999	2004
In vivo	2000	2.21	2000	2006
Contact hypersensitivity	2001	2.04	2001	2005
Apoptosis	2005	3.03	2005	2009
Betulinic acid	2006	2.01	2006	2008
Mechanism	2007	4.02	2009	2015
Photocarcinogenesis	2009	2.4	2009	2013
B16 melanoma cells	2009	2	2009	2010
Mushroom tyrosinase	2011	4.6	2011	2015
Polyphenols	2011	2.15	2011	2012
Model	2000	2.04	2011	2013
Pigmentation	2006	5.53	2012	2017
Biosynthesis	2005	2.53	2012	2014
Radical scavenging activity	1999	2.23	2012	2013
Activated protein kinase	2013	2.29	2013	2015
Children	2014	2.57	2014	2015
Vitamin C	2010	2.23	2014	2016
Exposure	2015	2.95	2015	2018
Human skin	2009	7.06	2016	2017
Melanogenesis	2008	5.21	2016	2017
Optimization	2016	2.88	2016	2018
Transcription factor	2012	2.32	2016	2017
Fibroblasts	2017	3.9	2017	2022
Natural products	2017	3.87	2017	2018
Toxicity	2015	2.87	2017	2018
TNF alpha	2017	2.01	2017	2019
Molecular mechanisms	2017	2.01	2017	2019
Sun protection factor	2018	5.2	2018	2022
Medicinal plants	2008	3.89	2018	2019
Aqueous extract	2014	2.53	2018	2019
Skin aging	2015	2.05	2019	2022
NF kappa B	2013	2.05	2019	2020
Wound healing	2020	4.83	2020	2024
Injury	2020	2.72	2020	2021
Quercetin	2017	2.69	2020	2022
Efficacy	2010	2.3	2020	2024
Release	2020	2.18	2020	2021
Protection	1999	2.07	2020	2021
Management	2019	3.51	2021	2024
Collagen	2018	3.35	2021	2022
Bioactive compounds	2010	3.12	2021	2022
Saponins	2016	2.4	2021	2022
Metabolism	2016	2.25	2021	2022
Design	2021	2.18	2021	2022
Caenorhabditis elegans	2022	2.95	2022	2024
Rosmarinic acid	2022	2.6	2022	2024
Economic growth	2012	2.51	2022	2024
Nanoparticles	2019	2.25	2022	2024
Assisted extraction	2022	2.17	2022	2024
Formulation	2015	2.16	2022	2024
Acetylcholinesterase	2013	2.1	2022	2024

*Note:* “Year” represents the time when the keyword first emerged. “Begin” represents the starting time when the keyword received explosive attention from the academic community. “End” represents the ending time of the explosive research period.

Beyond conclusions outlined in previous sections—such as the roles of natural herbs in skin pigmentation, antiaging, antioxidant activity, anti‐inflammatory effects, active compounds, extraction methods, and UV protection—we derived new insights from keyword bursts.

Keywords like “apoptosis,” “mechanism,” “molecular mechanisms,” “activated protein kinase,” “transcription factor,” and “NF‐kappa B” (involving cellular biology and signaling pathways) highlight sustained interest in mechanistic and molecular pathways linking natural herbs to skin health, with the NF‐κB‐related inflammatory pathway as a major focus. Combined with earlier‐stage keywords like “in vivo,” “B16 melanoma cells,” and “human skin,” this indicates that fundamental research on herbal skincare has evolved from in vitro to in vivo studies and from cellular to molecular‐level exploration.

Keywords such as “toxicity” and “children” reflect efforts to evaluate the safety, toxicity, and applicability of herbal ingredients in skincare, ensuring gentle formulations for sensitive skin and pediatric use.

Keywords such as “economic growth” and “management,” with burst periods persisting to the present, indicate that research in this field now extends beyond basic science to encompass economic and applied management aspects. Scholars are exploring the market potential and commercial value of natural herbal skin care products, investigating market size, growth rates, and consumer preferences, and proposing strategies for optimizing R&D and market deployment of herbal skin care products to uncover new commercial opportunities. The emergence of these keywords also underscores the importance of standardization and guidelines for dosage, usage, manufacturing, and ingredient composition of herbal formulations.

In product development and optimization, keywords like “optimization,” “assisted extraction,” and “formulation” reflect efforts to improve extraction techniques, bioactive ratios, and preparation processes for herbal skincare products. Innovations such as nanoparticle design (highlighted by “nanoparticles”) aim to enhance ingredient efficacy, skin permeability, stability, and bioavailability [44]. Meanwhile, design emphasizes user‐centric innovation and experience in skincare product engineering.

## 4 Discussion

In this study, we used the WoSCC database for a literature search that included 1593 published articles matching the topic. This research summarized the research hotspots and their changing trends in this field, and emphasized the importance of exploring the clinical efficacy and mechanism of traditional herbal medicine in the field of skincare. This research holds critical importance for understanding the components, targets, and mechanisms of traditional Chinese herbal medicine while driving the development and utilization of multifunctional, low‐toxicity, and highly selective herbal formulations. Active compounds derived from natural herbs show potential to prevent or delay skin aging and certain dermatological conditions, thereby reducing societal economic burdens. Over the past four decades—particularly since 2018—publications in this field have surged annually, peaking in 2024 with record output, a trend expected to continue. This reflects growing public recognition of herbal medicine in daily skincare, with research gradually shifting from macro‐level observations to microscopic molecular mechanisms. As the value of herbal extracts becomes increasingly evident, scholarly interest in exploring novel herbs and innovative applications will likely sustain long‐term growth in research output.

China leads in publication volume, a position tied to its rich cultural heritage and geographical diversity (spanning multiple latitudes, climates, and ecosystems). Chinese scholars have systematized the use of herbs in skin care for millennia, as documented in historical texts like the Compendium of Materia Medica (Bencao Gangmu), which details herbal formulations for skin whitening and other beauty purposes.

Institutional contributions mirror regional clustering, with nearly all top 20 research institutions based in East Asia, underscoring the field's geographical specificity. However, collaboration among authors remains limited, as indicated by centrality values near zero. This may stem from traditional research practices, such as regional variations in herbal knowledge, mentorship‐based knowledge transfer, and formula confidentiality, as well as the early‐stage dynamics of emerging interdisciplinary fields, where pioneers often work independently before collaborative networks mature.

In herbal skincare research, *Industrial Crops and Products*—with the highest citation count—is the most influential journal, followed by the *Journal of Ethnopharmacology* and *Molecules*. These journals, along with others like *EMBO Journal* and *Biochemical Society Transactions*, highlight the intersection of pharmacology, biochemistry, and translational research, driving multidisciplinary advancements in the field.

Traditional Chinese medicine research is completing the transition from “experience” to “evidence‐based.” In the beginning, ancient physicians relied on the combination of ancient texts and practical experience in the use of herbs for skin whitening and anti‐aging treatments. With advances in science and technology, researchers are eager to gain a deeper understanding of the mechanisms of action of herbs. During the formative stage (2000–2005), investigations predominantly focused on botanical screening through primitive extraction methods (aqueous/ethanolic extracts [45, 46]) and phenotypic validation using animal models. This phase established foundational evidence for herbal efficacy in UV protection (75% UVB absorption rate for green tea polyphenols [23]) and pigment inhibition (45% tyrosinase activity reduction by licorice extracts [47]). However, the reductionist approach limited mechanistic insights, as evidenced by keyword clusters (#19 and #0) [47] dominated by “crude extract” and “in vivo.”

The subsequent mechanistic expansion phase (2006–2015) witnessed paradigm‐shifting advancements through precise cellular and animal models to elucidate the skin care mechanisms of traditional herbal medicine, and gradually transitioned from the “cellular” to the “molecular” level. An empirical Chinese herbal preparation, called Shi Zhen Formula (SZF), whose potential molecular mechanism in atopic dermatitis is well reflected by Lan Wang et al., demonstrated the inhibition of NF‐κB p65 pathway activation using ex vivo modeling, thereby improving the epidermal appearance of dermatitis caused by atopic dermatitis. This improves epidermal dysfunction caused by dermatitis and promotes skin health [48]. Models have been systematically summarized, and the development of the system has tended to mature [49]. Research on the therapeutic properties of herbal cosmetics will continue to evolve, accelerating the influx of studies on the mechanisms of action of herbs. Researchers have further validated that herbs can reduce inflammation and cellular damage by modulating signaling pathways, specifically the NF‐κB, MAPK, PI 3 K/AKT, and Nrf 2/ARE pathways, to achieve delayed skin aging [50, 51, 52, 53]. On the other hand, the natural compounds promote the TGF‐β pathway, inhibit MMP activity, and enhance collagen synthesis, while possibly modulating the mTOR pathway, thus protecting the dermal collagen network and highlighting good anti‐wrinkle efficacy [54, 55]. For example, ginseng, a famous and valuable Chinese herb, has an active ingredient that increases the activity of antioxidant enzymes in skin cells and inhibits the formation of ROS [56].

Adding herbal extracts to skincare requires a better understanding of the potential of herbs. In conclusion, the understanding of the action modes of traditional herbal medicine has changed from a single‐target paradigm to one involving multi‐ingredient, multitarget, and multipathway. Traditional herbal medicines exert therapeutic effects mainly through formulations composed of different kinds of chemical ingredients. This suggests that different active ingredients are likely to target different pathways, and the compatibility among these active ingredients allows traditional herbal medicines to care for the skin from a holistic perspective. However, these ingredients include active ingredients, inactive ingredients, and even toxic substances, which have unpredictable dose‐responses and toxicological properties [57]. Therefore, the separation and identification of key active ingredients from traditional Chinese medicines, the research of their mechanism of action, compatibility, and potential toxicity, and the exploration of the optimal ratio of different active ingredients are the basis for improving the formulation of traditional Chinese medicines, improving the utilization efficiency of monomers, and designing multitarget, low‐toxicity drugs to regulate skin problems. This method not only conforms to the holistic and dialectical therapeutic principles of traditional herbal medicine but also reflects the precision medicine concept of modern medicine, breaking the traditional thinking of “one drug, one target, one disease.” The integration of network pharmacology with various types of analytical techniques provides new insights into the complex study of traditional herbal medicines. Mass spectrometry can be adapted to screen and isolate specific bioactive chemical components in traditional herbal medicines [58]. Molecular docking techniques can simulate and verify interactions between the ingredient targets and gout targets predicted by network pharmacology [59]. Molecular similarity analysis can reveal the synergistic relationship among different active ingredients and determine the rationale behind the combination of medicinal ingredients.

The Chinese government, driven by the goal of preserving traditional culture, is progressively integrating traditional medicine into the healthcare system [60, 61]. As a complementary and alternative approach to skincare, herbal therapies offer broad applicability and significant economic benefits, making the market potential and commercial value of natural herbal skincare products a focal point for scholars. American dermatologist Albert Kligman pioneered the concept of “cosmeceuticals”—products that blend cosmetic and pharmaceutical properties—which is emerging as a new frontier for traditional herbal medicine in skin care. While international regulatory frameworks lack uniformity for such products, the term “cosmeceutical” is widely recognized and utilized within the industry [16, 62, 63]. For example, Japan has established a regulatory category called “quasi‐drugs” for these products, requiring specific approvals. To unlock market opportunities, scholars emphasize the need to analyze market size, growth rates, and consumer preferences while developing effective R&D and marketing strategies for herbal skincare products. Increased scientific investment is essential to produce evidence‐based, high‐efficacy formulations. Brands should also prioritize consumer education, enhancing public understanding of product benefits and ingredients to build trust. Regulatory bodies must enforce standardized guidelines for dosage, usage, manufacturing, and ingredient composition of herbal formulations, as a unified regulatory framework would advance systematic development and global market integration of cosmeceuticals [24]. Overall, the use of herbal ingredients in cosmetics has grown significantly. Current research focuses heavily on preclinical studies exploring plant‐based solutions for skin improvement. Future efforts should prioritize multicenter clinical trials and evidence‐based medical research to validate the efficacy and safety of herbal products, laying the groundwork for their global adoption [64].

## 5 Conclusion

This study conducted a comprehensive literature search in the Web of Science Core Collection (SCI‐Expanded index), yielding 1593 records (1981–2024). Using visualization tools including CiteSpace, VOSviewer, and ArcGIS, it analyzed leading countries, institutions, authors, journals, research hotspots, and evolutionary trends in herbal skincare. The analysis decoded the research landscape and innovation patterns of the field, systematically summarizing over four decades of development trajectories, hot topics, current status, and future directions. This study provided a framework for integrating traditional herbal wisdom with modern medical science, proposed key future research directions in herbal skincare, identified scientifically or economically promising entry points, and highlighted existing challenges in the field.

## Author Contributions

Conceptualization: K.D., Y.L.; Methodology: K.D.; Formal analysis: C.L.; Investigation: C.L., Y.L.; Writing – original draft: K.D., Y.L.; Writing – review and editing: K.D., D.L.; Resources: C.L.; Supervision: D.L.

## Ethics Statement

The authors have nothing to report.

## Conflicts of Interest

The authors declare no conflicts of interest.

## Supporting information


Data S1.

## Data Availability

Data sharing is not applicable to this article as no new data were created or analyzed in this study.
