# Supplementary material for: Applications of Traditional Herbal Ingredients in Skincare: Mapping the Research Landscape and Innovation Trajectories Over Four Decades
Source: J Cosmet Dermatol. 2025 Jul 21;24(7):e70363. doi: 10.1111/jocd.70363 (PMC12278037; doi:10.1111/jocd.70363)
Supplement: Supplementary file 1 — Data S1. [file JOCD-24-e70363-s001.docx]

CiteSpace is an information visualization tool based on citation analysis theory developed by Dr. Chen Chaomei from Drexel University. It can explain the structure, patterns, and distribution of scientific knowledge, producing the so-called “scientific knowledge graph”[1]. CiteSpace is mainly used to organize theoretical viewpoints, track evolutionary paths, predict development trends, conduct in-depth research on academic history, and identify current research hotspots within a particular field[1]. It is a practical quantitative analysis tool used to scrutinize research documents[2].

CiteSpace analysis methods include co-citation analysis, co-occurrence analysis, burst detection, and cluster analysis[1]. Co-citation analysis consists of examining the relationship between two studies based on their co-citation in the third study. Studies that are frequently cited together are considered to be more similar and interrelated. Co-occurrence analyzes the number of times that a particular keyword appears in the literature of a given field and measures the correlation between them by co-occurrence. Burst detection can identify fluctuations in the use of particular keywords, while cluster analysis groups object on the basis of their similarities, helping to analyze multiple clusters formed[3-6].

Centrality is a critical metric that gauges the importance of an object in a network. Nodes with an intermediate centrality value greater than 0.1 are called central nodes or key nodes, which have an important influence in the research field and are often act as bridges connecting different kinds of research objects like articles, keywords, and countries[7].

CiteSpace is uniquely positioned to pinpoint pivotal aspects and future directions in the field of research, making it an invaluable tool for scholarly investigations[1]. In this study, the bibliometrics analysis software CiteSpace was deployed to analyze the existing literature on herbal medicine for skincare. Critical readings were also conducted to delve into key research findings and provide vital insights into this subject matter.

1. Chen, C.M., *CiteSpace II: Detecting and visualizing emerging trends and transient patterns in scientific literature.* Journal of the American Society for Information Science and Technology, 2006. **57**(3): p. 359-377.

2. Hood, W.W. and C.S. Wilson, *The literature of bibliometrics, scientometrics, and informetrics.* Scientometrics, 2001. **52**(2): p. 291-314.

3. Donthu, N., et al., *How to conduct a bibliometric analysis: An overview and guidelines.* Journal of Business Research, 2021. **133**: p. 285-296.

4. Ding, Y., G.G. Chowdhury, and S. Foo, *Bibliometric cartography of information retrieval research by using co-word analysis.* Information Processing & Management, 2001. **37**(6): p. 817-842.

5. Su, H.N. and P.C. Lee, *Mapping knowledge structure by keyword co-occurrence: a first look at journal papers in Technology Foresight.* Scientometrics, 2010. **85**(1): p. 65-79.

6. Liu, Z.G., et al., *Visualizing the intellectual structure and evolution of innovation systems research: a bibliometric analysis.* Scientometrics, 2015. **103**(1): p. 135-158.

7. Wang, L.Y., et al., *Way to accomplish low carbon development transformation: A bibliometric analysis during 1995-2014.* Renewable & Sustainable Energy Reviews, 2017. **68**: p. 57-69.
